# An integrated approach for increasing breeding efficiency in apple and peach in Europe

**DOI:** 10.1038/s41438-018-0016-3

**Published:** 2018-03-01

**Authors:** Francois Laurens, Maria José Aranzana, Pere Arus, Daniele Bassi, Marco Bink, Joan Bonany, Andrea Caprera, Luca Corelli-Grappadelli, Evelyne Costes, Charles-Eric Durel, Jehan-Baptiste Mauroux, Hélène Muranty, Nelson Nazzicari, Thierry Pascal, Andrea Patocchi, Andreas Peil, Bénédicte Quilot-Turion, Laura Rossini, Alessandra Stella, Michela Troggio, Riccardo Velasco, Eric van de Weg

**Affiliations:** 1 IRHS, INRA, Agrocampus-Ouest, Université d’Angers, SFR 4207 QuaSaV, Université Bretagne Loire, 42 rue Georges Morel, Beaucouzé, 49071 France; 2 IRTA (Institut de Recerca i Tecnologia Agroalimentàries), Barcelona, Spain; 3 Centre for Research in Agricultural Genomics (CRAG) CSIC-IRTA-UAB-UB, Campus UAB, Bellaterra, Barcelona Spain; 4 Università degli Studi di Milano - DiSAA, Via Celoria 2, Milan, 20133 Italy; 5 Biometris, Wageningen University and Research, Droevendaalsesteeg 1, Wageningen, 6708PB The Netherlands; 6 IRTA-Mas Badia, Mas Badia, La Tallada, 17134 Spain; 7 Parco Tecnologico Padano, Via Einstein, Loc. Cascina Codazza, Lodi, 26900 Italy; 8 Dipartimento di Scienze Agrarie, Alma Mater Studiorum - Università di Bologna, Bologna, Italy; 9 UMR AGAP, INRA, Montpellier, 34398 France; 10 GAFL, INRA, Montfavet, 84143 France; 11 Agroscope, Research Division Plant Breeding, Schloss 1, Wädenswil, 8820 Switzerland; 12 Julius Kühn-Institute (JKI); Federal Research Centre for Cultivated Plants, Institute for Breeding Research on Fruit Crops, Pillnitzer Platz 3a, Dresden, 01326 Germany; 13 Research and Innovation Center, Fondazione Edmund Mach, San Michele all’Adige, Trento, Italy; 14 CREA-VE, Center of Viticulture and Enology, via XXVIII Aprile 26, Conegliano (TV), 31015 Italy; 15 Plant Breeding, Wageningen University and Research, Droevendaalsesteeg 1, P.O.Box 386, Wageningen, 6700AJ The Netherlands; 16 Present Address: Hendrix Genetics Research, Technology & Services, Boxmeer, 5830 AC The Netherlands

## Abstract

Despite the availability of whole genome sequences of apple and peach, there has been a considerable gap between genomics and breeding. To bridge the gap, the European Union funded the FruitBreedomics project (March 2011 to August 2015) involving 28 research institutes and private companies. Three complementary approaches were pursued: (i) tool and software development, (ii) deciphering genetic control of main horticultural traits taking into account allelic diversity and (iii) developing plant materials, tools and methodologies for breeders. Decisive breakthroughs were made including the making available of ready-to-go DNA diagnostic tests for Marker Assisted Breeding, development of new, dense SNP arrays in apple and peach, new phenotypic methods for some complex traits, software for gene/QTL discovery on breeding germplasm via Pedigree Based Analysis (PBA). This resulted in the discovery of highly predictive molecular markers for traits of horticultural interest via PBA and via Genome Wide Association Studies (GWAS) on several European genebank collections. FruitBreedomics also developed pre-breeding plant materials in which multiple sources of resistance were pyramided and software that can support breeders in their selection activities. Through FruitBreedomics, significant progresses were made in the field of apple and peach breeding, genetics, genomics and bioinformatics of which advantage will be made by breeders, germplasm curators and scientists. A major part of the data collected during the project has been stored in the FruitBreedomics database and has been made available to the public. This review covers the scientific discoveries made in this major endeavour, and perspective in the apple and peach breeding and genomics in Europe and beyond.

## Genomics: New resources for apple and pear breeding

A Europe-led effort to bridge the gap between genomics and breeding offers a new resource to support apple and peach production. A team led by François Laurens from the Research Institute in Horticulture and Seeds (IRHS) in Angers, France, review the tools, methodologies and scientific discoveries made through FruitBreedomics, a project that ran between 2011 and 2015 and involved 28 different research and private companies from around the world. These new molecular and bioinformatics tools include DNA arrays for mapping genetic diversity, computer software for analyzing genetic information and novel methods for understanding commercially important traits such as fruit quality and resistance to stresses. The project’s materials should help apple and peach producers better plan their breeding programs so as to meet grower and consumer demand for high-quality fruit with desired characteristics.

## Introduction

Fruit industry is facing economic challenges imposed by increasingly fierce international competition and decreasing fruit consumption, societal demand for a more sustainable production, and biological problems caused by climate changes. Releasing new cultivars that meet these challenges is a major goal of all breeding programs. For decades, response to address them has been slow due to the nature of fruit tree breeding: long term; low efficiency; and hence high cost. For the past 20 years, international networks of fruit geneticists and genomicists have been built to develop research on fruit genetics aimed at enhancing fruit quality traits as well as resistance to biotic stresses. The commonly applied aim of these programs was to release molecular markers associated to major agronomic traits to help and improve the efficiency of the breeding programs.

Despite all these efforts, in 2010 very few breeding programmes were adopting the output of these researches. From this premise, a European consortium listed the main bottlenecks that hampered the use of new technologies in breeding programmes^1^: at this time, the most common molecular markers used to build fruit genetic maps were SSRs^2,3^; their high quality and polymorphism had made them very helpful for diversity studies but high cost and low throughput made them unsuitable to build dense genetic maps; this resulted in big gaps in genetic maps and limited resolution in quantitative trait loci (QTL) mapping. In addition, all the genetic studies were based on bi-parental progenies leading to a lack of information on the genetic diversity which was practically used in commercial breeding programs^3^. To overcome these challenges, the European Union thanks to the Seventh Framework Programme (2007–2013) funded the FruitBreedomics consortium from March 2011 to August 2015 to take advantage of the first initiatives to decipher fruit species genomes (first genome sequence of apple^4^; Peach Genome Sequencing Initiative^5^). This European large collaborative project aimed at making use of the latest methodologies, technologies and knowledge to improve the efficiency of current fruit breeding programmes. It focused on apple and peach, two major fruits in Europe, keeping in mind that many tools and much knowledge gained could also be of benefit to other species of the Rosaceae family via the strong genomic and functional ancestral relatedness among these species.

FruitBreedomics, with researchers from 28 research institutes and private companies (Supplemental Table 1) had a goal of bridging the gap between genomics and breeding in fruit trees. Three main approaches were pursued: (1) developing tools/softwares/methodologies; (2) deciphering genetic control of main agronomical traits taking into account allelic diversity; and (3) developing plant materials, tools and methodologies for the breeders.

## Development of phenotypic and genotypic tools

After the completion of two surveys, one for apple and one for peach, in which stakeholders as well as partners of the project were asked to describe their breeding programs and breeding aims, scientists were able to identify the most important traits for apple and peach breeders. In the case of apple the top five traits in breeding were: scab resistance; storability; juiciness; crispness; and firmness. For peach the most important traits were: fruit size; homogeneity and type; juiciness; crispness and sweetness; and finally brown rot, mites and green peach aphid resistance. The traits that have been prioritized by the stakeholders are in good agreement with the traits that have been selected for genetic studies in FruitBreedomics. The same survey also allowed having a first insight on how apple and peach are currently bred and selected.

### Developing tools for phenotyping main horticultural traits

Taking into account these surveys and new consumer trends, global climate change, environmental concerns and consultation with stakeholders, FruitBreedomics decided to develop new phenotyping tools for attributes of key importance for the fruit industry, i.e., fruit texture and resistance for some biotic and abiotic stresses for which no simple, reliable and high-throughput assessment methods were previously available.

#### Fruit texture

In apple, fruit texture is a complex of several components, fundamentally divided in two main groups: mechanical (such as firmness, gumminess, hardiness, etc.) and acoustic (crispness and crunchiness). A TA-XTplus Texture Analyzer equipped with an Acoustic Envelop Detector (AED) device (Stable MicroSystem Ltd., Godalming, UK)^6^ was used to acquire simultaneously the acoustic-mechanical profile of apple texture and thus perform a more detailed investigation of each single component including crispness.

In peach, slow melting flesh (SMF) varieties, like ‘BIG TOP’ have become de ‘facto’ standard in the industry because of their high fruit quality and better handling after harvest. However, this trait, characterised by a slower firmness loss than melting flesh fruit after harvest, is quite difficult to phenotype. Two methodologies tested in FruitBreedomics showed promising results: (i) time-course postharvest evaluation with a hand-held penetrometer (ii) rheograms acquired from digital penetrometer (Fruit Texture Analyzer, FTA, RG strumenti). Both of them are now recommended to breeders who develop this type of varieties. Two peach progenies having ‘Big Top’ as one of the parents were genotyped with the 9K single-nucleotide polymorphism (SNP) chip (see below) and phenotyped with a hand-held penetrometer at different stages of the postharvest process. After mapping two major independent QTL were detected, one exclusively found in ‘Big Top’^7^. Specific alleles at these two loci explained each >20% of the variability for SMF and this trait was, in turn, associated with maturity date, late maturing individuals having an increased SMF. A third unlinked QTL was also found that affected maturity time but not flesh texture, allowing for genotype combinations that are early maturing and have the SMF phenotype^7^.

#### Resistances to biotic stresses

Peach brown rot caused by *Monilinia spp*. induces severe damage in the orchard but also during transport and storage^8^. Efforts outstretched during the life of the project participated in compiling the information currently available on this disease^9^, developing new artificial infection assessment to screen progenies and exploring fruit physical and biochemical characteristics as potential resistance factors (publication submitted). Specifically, surface compounds, waxes and epidermis phenolic compounds may contribute to the resistance of the fruits in the middle stage of growth (pit hardening) (upcoming publication). Though environmental control may be important, the variability of susceptibility to *M*. *laxa* and fruit characteristics observed within progenies indicated a quantitative inheritance of these traits, further studied via QTL analyses^10^. Some genotypes that displayed infection probability equal to zero for different years, sites or tests are currently used to develop peach cultivars resistant to brown rot.

#### Resistances to abiotic stresses

**Fig. 1:**
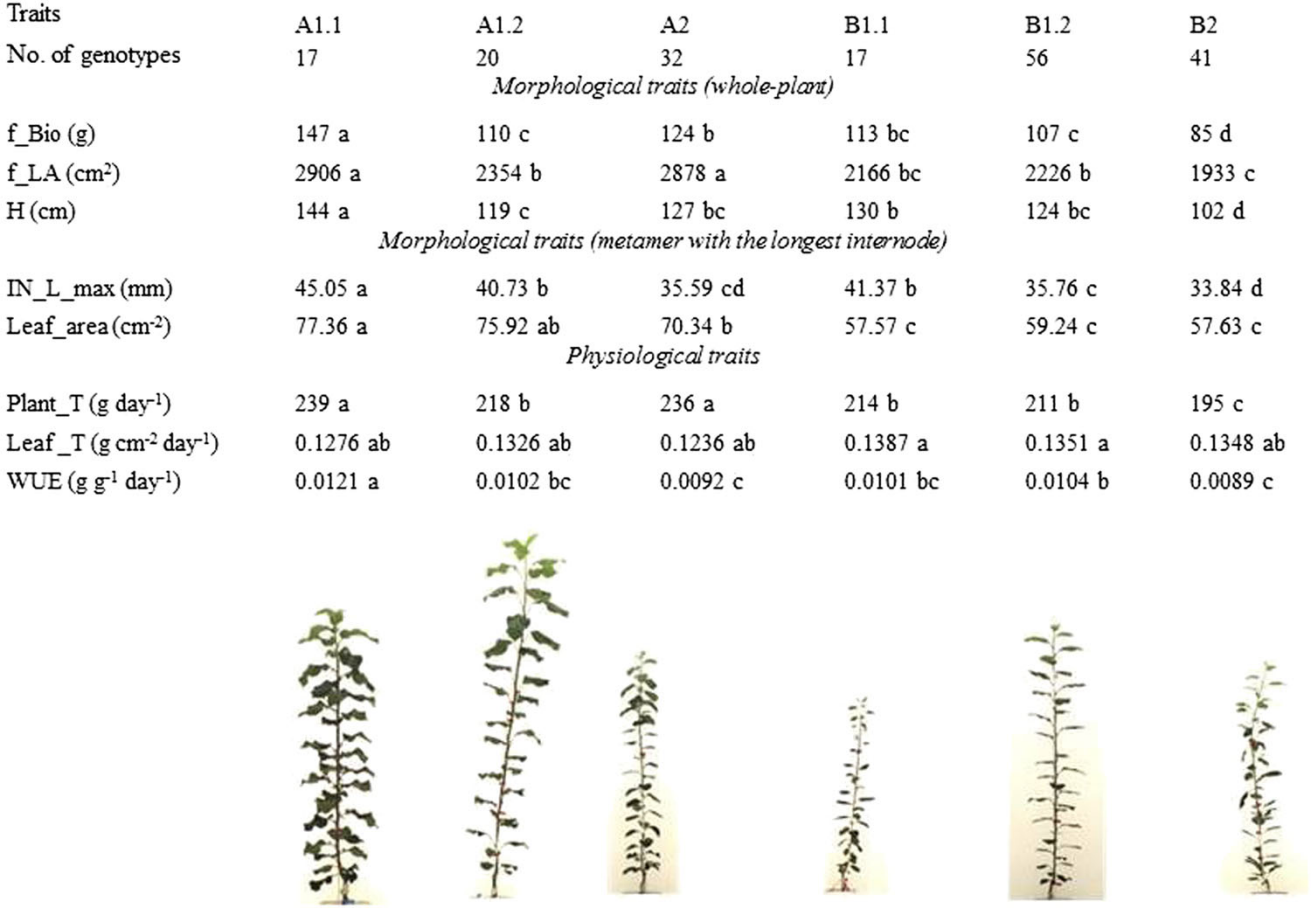
Illustration of the six clusters obtained from a hierarchical ascendant classification (HAC) of an apple core collection based on morphological traits measured. Information of the physiological traits and one characteristic genotype for each group are also presented (adapted from Lopez et al. 2015^12^)

Global climate change predictions indicate decreased water availability in many important fruit growing areas. Selection of apple and peach cultivars that might endure water stress is going to be in the ‘to do list’ of fruit breeding programs as it is in other plant species. However, the assessment of water stress resistance is neither simple nor fast. FruitBreedomics has explored two methods to assess tolerance/resistance to water scarcity. First, a multivariate statistical approach was developed to assess leaf photo-assimilation performance using a portable photosynthesis system. Water stress tolerance of genotypes could be discriminated by the IPL index, correlated with photosynthetic activity and variables representing their morphological responses^11^. Second, the role of plant morphology was explored using high-throughput phenotyping technologies (PhenoArch platform). For the first time in fruit trees, the variability of water use efficiency (WUE) and the respective roles of plant biomass and transpiration were explored within a French core collection. The total plant biomass was highly correlated to WUE, and two of its components (maximum internode length and plant height) were highly heritable. Groups of morphotypes were identified based on organogenesis and organ dimensions (Fig. 1) and were linked to different WUE^12^. On a subset of genotypes issued from a bi-parental population, the effects of two consecutive periods of soil drought, moderate and severe, were assessed on growth and functional characteristics of leaf and stem. Both reduction of leaf area and leaf organogenesis appeared key determinants for drought avoidance in apple, with strong genotypic variations. The pivotal role of stomatal conductance in maintaining ‘percent loss of conductivity’ of the stem xylem (PLC) under severe drought was confirmed^13^. The variability among genotypes of morphologic and functioning responses to soil water contents illustrates a promising genetic diversity and opens the way for improving apple plant materials for the use of water.

Besides water availability concerns, erratic phenology may also hamper production in some regions in the future. Besides forcing tests that proved relevant to select cultivars with desired chilling and heating requirements, phenological models that requires observed data easier to screen allowed to progress in the dissection of budbreak and flowering time genetic architecture^14^. Objectives may focus on the selection of cultivars with either lower chilling requirements or higher heat requirements of the floral buds to prevent from spring frost risk or excessive flowering advances.

### Implementation of bioinformatics tools to store and access to the data of the project

Bioinformatics infrastructures were designed and deployed to provide efficient storage and access of phenotypic data and genome sequence variation information. We developed and managed web-based relational data banks specifically created for apple and peach data, and provided specific bioinformatics tools for data integration and visualization^15^. All data are accessible through a stable and well-maintained interface, available at the address http://bioinformatics.tecnoparco.org/fruitbreedomics.

The database contains a shared nomenclature for phenotypes attributes and descriptors, and a list of consensus cultivar names from several countries. It also stores genotypes and phenotypes collected during several years of observations on collections of plants, characterised by cultivars and geographical locations. Two separate database systems have been developed, along with their two interfaces. The first one stores phenotype data relying on an experiment-based model. This database reflects, in its structure, the natural data shape of the phenotyping information: trees, experimental orchards, phenotypes and genotypes are represented as separate and interconnected tables, in a classic relational data model; this facilitates standardization and importing/integration procedures, as well as update of this particular type of data. While an automatic procedure allows to upload large amounts of data, performing data validation in the meantime, a second interface, constituted by web pages containing modules and forms, allows researchers to manually input information (ensuring integrity and correctness of data) and to surf and update database content. All the information included in this part of the system is periodically copied, together with other types of data (e.g., genotypes) to the final database, into a Chado-like structure (http://gmod.org/wiki/Chado), the same underlying many installations including The Genome Database of Rosaceae (GDR—http://rosaceae.org/). With this infrastructure, Fruibreedomics data landscape were aligned to GDR, easing data exchange between the two projects.

On top of this Chado database, a Drupal/Tripal interface has been developed, integrating several tools. Two are the objectives of this web application: to leverage the interconnections between several separated datasets collected in the FruitBreedomics database, and to provide the functionalities to easily perform analyses. A particular effort was devoted to Linkage Disequilibrium Analysis (LD), and Marker Based Analysis (MBA), with the development of two custom online tools, namely *Ldexplorer* and The *Breeder’s Interface*. The web interface allows an easy access to genetic data from SNP arrays, SSR analysis, and haploblock inference. Moreover, it connects the genotyped samples to collected phenotypes. Each sample refers to a tree, whose genotype is recorded, with information on pedigree stored as a network of connections between genotypes. The *LDexplorer* and *Breeder’s Interface* tools respond to the explicit requirements of the scientists/breeders community. The tools are completely integrated in the website. The Breeder’s Interface is a tool aiming to help breeders to plan crosses. It integrates information coming from years of phenotypic and marker data gathering, and implements the requirements for MBA. *LDexplorer* facilitates linkage disequilibrium analyses on SNP array data. It provides both numeric and graphical results.

### Implementation of SNP arrays for fine mapping

At the beginning of the project, three DNA arrays were available: one for peach with 9K SNP markers from the IPSC (International Peach SNP Consortium)^16^ and one for apple with 384 SNPs used for gene diversity and linkage mapping studies^17^, followed a few months later by a 8K SNP markers produced by the IRSC (International RosBreed SNP Consortium^18^). The FruitBreedomics consortium, after evaluation of the efficiency of the apple SNP arrays available, decided to develop two higher density chips for apple in order to fulfil the objectives of the project in particular to fine map QTLs for main horticultural traits. Firstly, a new array with a target size of 20K SNPs, was developed in order to better cover chromosome ends and other gaps within chromosomes. A number of robust SNP markers (3.7K) from the available IRSC apple 8K Infinium SNP array were included in the new 20K Illumina Infinium array, while an additional 16.3K SNPs were predicted from re-sequencing data derived from 14 apple genotypes and two double-haploids from ‘Golden Delicious’^19^. A haplotype-targeting strategy similar to that adopted to design the IRSC array was implemented for the design of the array, in order to combine the information from individual SNPs into haploblocks and provide fully informative multi-allelic markers. An ad hoc pipeline for SNP selection was devised to reach the target of 20K SNPs, while at the same time avoiding the pitfalls presented by paralogous sequence variants. The performance of the SNP array was assessed using data from >2000 seedlings from 25 full-sib families^14,19,20^ and ~400 cultivars and breeding selections. This evaluation revealed that >75% of the SNP assays were polymorphic and segregated in a Mendelian fashion. The genetic linkage maps constructed on individual families had an average of 6.8K SNPs uniformly distributed along the genome^19^, which provided ~7000 high-quality markers suitable for pedigree-based QTL mapping on multi-parent populations (see below).

The genetic position of ca. 16K SNPs was used for the re-ordering of contigs from the first apple genome sequence into an improved version of the apple genome (v3.0) now available in a recent de novo assembly of a ‘Golden Delicious’ doubled-haploid whole genome sequence^21^ both of which are available through the GDR databasewww.rosaceae.org. Using the information obtained from the 20K array on wide germplasm it was also possible to study the decay of linkage disequilibrium (LD) for each apple chromosome. As expected, LD decays quite rapidly, at an average distance of 55 kb (personal communication). A much higher density array was therefore necessary to perform Genome Wide Association studies (GWAS) in apple.

Thus, a 487K SNP array, the largest ever produced for a fruit plant and among the largest for higher plants, was developed using Affymetrix technology^22^. For SNP discovery a high coverage whole-genome re-sequencing strategy across a panel of highly diverse apple cultivars was adopted to call individual genotypes and reduce the number of false-positive SNPs included in the array design. This also allowed the selection of sets of polymorphic markers that together represented the diversity of the discovery panel. All the robust SNP markers from the previous 20K Illumina Infinium array were included together with 1.5K SNPs derived from a Genotyping By Sequencing experiment, while the remaining 465K SNPs were newly predicted from a re-sequencing of 63 apple genotypes and two doubled haploids (available upon request). The Axiom Apple480K Chip was successfully produced and it is now public available. Using a quality prediction model based on logistic regression, a total of 275,223 highly informative and robust SNPs (56% of the 487K) were identified^22^. This newly developed array was used for apple GWAS on 1324 apple accessions from six European germplasm collections and for the construction of four high-density parental maps^23^.

For peach, the IPSC (the International Peach SNP Consortium) 9K SNP array was successfully used for genotyping both segregating populations and germplasm collections. The average space between SNPs in the array is 26.7 kb, which corresponds to 16.5 SNPs per cM^16^. Since LD in peach was estimated at about 13–15 cM^24^, the density of the array is sufficient to perform optimal GWAS in peach with the goal of obtaining SNP closely linked to horticultural traits of interest. Among the 8144 included in the array, 4271 were scored as polymorphic in a collection of 1580 peach accessions (including an ample set of Occidental and Oriental materials) and an average of 2166 validated SNPs were obtained for each of 18 peach progenies (derived from intra- and interspecific crosses). In addition, the genotyping of several segregating progenies, allowed the construction of dense SNP maps for accurate QTL detection, as demonstrated by a first application for the identification of QTLs for volatile organic compounds involved in peach fruit aroma^25,26^.

## Improving knowledge on the genetics of main traits

The second main aim of FruitBreedomics was to improve knowledge on the inheritance of important traits and to discover and characterize marker trait associations that could be made applicable for MAB through the development of DNA diagnostic tools. These genetic studies have been performed thanks to two complementary strategies both based on examining and exploring a wide genetic diversity: the Pedigree Based Analysis (PBA), which is based on the integrated analysis of multiple pedigreed full-sib families^27,28^ and GWAS based on the analysis of populations of unrelated cultivars. Both approaches aimed to decipher the genetic control of major agronomic traits and provide DNA diagnostic tools to support breeders in the selection of new cultivars.

### PBA

#### New software

Software packages were developed to provide an efficient workflow for genetic studies using SNP markers from the filtering and calling of markers through the phasing of SNP marker alleles, the building of haploblocks and haplotypes to PBA-based QTL discovery software. Firstly, the ASSIsT software allows a smooth, fast and reliable filtering and calling of Infinium derived SNP markers. The format of its input files are compatible with output files from GenomeStudio (Illumina Inc., San Diego, CA, USA; http://www.illumina.com)^29^.

FruitBreedomics successfully extended the software FlexQTL for use on SNP markers. FlexQTL was developed in the previous EU-HiDRAS project^30^ to enable multi-family analyses and was ready for the use of SSR markers^27^. As thousands SNP markers can now be tested for less than the price of a few SSR marker, sizes of datasets increased dramatically. On the other hand, single SNP markers are more difficult to use on complex germplasm because single markers are less informative. Thanks to collaborative efforts between FruitBreedomics and RosBREED^31^, FlexQTL was successfully adapted for application to high density SNP data sets, which included increased phasing performance for SNP data. Also its user friendliness has been considerably improved through the development of the Graphical User Interface Visual FlexQTL which provides support in the creation of input files, the running of the FlexQTL software itself, and for a structured examination of the FlexQTL output. Both softwares are publicly available at www.flexqtl.nl.

To further enhance the applicability of SNP data in genetic analyses, and dramatically reduce computation time of QTL discovery studies, the PediHaplotyper software was developed^32^. It compresses the information from sets of SNP markers into a single, virtual, multi-allelic haploblock marker, thus providing a much smaller data set without giving in information. These compressed data require less computation time and computer memory and are also more amenable for visual inspection and for a visual search for close and distant genetic relationships^32^, PediHaplotyper makes use of linkage phase and recombination information from FlexQTL output, and of functionalities in VisualFlexQTL for Haploblock sizing,

These new PBA softwares are publicly available and have already been used in a series of studies^14,33–47^.

#### Genetic linkage maps in apple and a new mapping approach

The accuracy by which gene positions can be localised is affected by many factors, including the order of the markers used in the analyses. Ideally, this order is identical to their true order on the chromosomes. For peach, this order can be adequately deduced from the known reference genome, due to the high quality of the sequence, which was further improved also with the use of genetic maps produced in the FruitBreedomics project^48,49^. For apple, this approach could not be followed as use of the available version of the genome sequence resulted in too many mismatches. Therefore, a new, high quality genetic linkage map was created on which >16K markers of the 20K array were ordered^20^. New innovative approaches were used in the map creation to overcome issues (i.e., large homozygous regions, missing values, variation in recombination intensity among parents) that usually have a major negative impact on map quality. This included the use of multiple full-sib families (21), reduction in the amount of missing data through a bin-mapping strategy, and the transformation of the data from a cross pollination design to a back cross design, as outlined by Di Pierro et al.^20^. The dedicated software HapAg was developed in support to this approach, which has been made public available at http://www.wageningenur.nl/en/show/HaploblockAggregator.htm.

Next, this map was used in FruitBreedomics for PBA based QTL discovery studies and in further works to improve the apple reference genome sequence^21^ as a reference for the completion of other genetic linkage maps in apple^38,47^.

#### New SNP markers in peach

In peach, the ability to position a target gene to a very confined region is regularly limited due to shortage of informative markers. Here, informative means that a marker reveals a difference (polymorphism) between two homologous chromosomes. In FruitBreedomics over 1.3 million new polymorphisms have been identified by examining the genome sequence of four important parents through a re-sequencing experiment. Next, some new SNP markers were designed and applied on some specific families, allowing to narrow down the potential position of a ‘Maturity Date’ gene by 27% to only 159 kb. The new polymorphisms have also been used for the design of an higher density Illumina peach SNP chip (18k peach SNP chip) in the context of an international collaboration involving FruitBreedomics and RosBREED partners (personal communication).

#### Marker-trait associations & predictive markers

In peach seven key horticultural traits (flowering and maturity date, fruit development period, percentage of red overcolor on the fruit skin, titratable acidity, soluble solid content and fruit weight) measured over several years were analysed in 18 progenies (1467 plants) from five European breeding programs. These were located in INRA-Avignon (France), INRA-Bordeaux (France), IRTA-Lleida (Spain), MAS.PES program (joint project between UMIL-Milan and CRPV-Cesena, Italy) and CREA-Rome (Italy). The progenies derived from both commercial and non-commercial parents (from peach or peach related species). As no individuals were duplicated or common between locations, to remove the effect of the environment in the analysis, phenotypic data was standardized. The progenies were genotyped with the 9K IPSC array. After filtering the SNPs for quality as described in the ASSIsT pipeline, polymorphic SNPs were grouped in haploblocks of 1 cM. Phenotypic (including raw and standardized data) and genotypic data were analysed with PBA software. We identified 47 QTLs, 22 coinciding with major genes and QTLs found in previous analyses and 25 new. Nearly half of the QTLs (47%) were detected only when analysing non-commercial progenies. This strategy also provided estimations on the narrow sense heritability of each trait and the estimation of the QTL genotype and breeding value of each parent^43,50^.

In apple a series of fruit quality traits have been studied on 25 mapping populations from seven breeding programs from six European countries for which the phenotypic data were collected as part of the previous EU-HiDRAS^51^ and EU-ISAFRUIT and some national projects. In FruitBreedomics, this corresponding germplasm has been genotyped with the 20K SNP array^19^. Marker trait associations have been found, and their stability over storage have been evaluated. Papers to report on this in detail are in progress for fruit firmness and storability, acidity and total sugars, fruit size, fruit color, and pre-harvest fruit drop. Presentations on part of the results on these are accessible through the FruitBreedomics website. Findings on the genetics of budbreak, flowering time and regular bearing have been published^14,44^.

#### Pedigree records: use, validation, and reconstruction

The use of marker information on pedigrees increases the power of PBA-based QTL discovery^28^ and facilitates composing germplasm sets for PBA studies^52^. This information is also useful in clarifying the origin and mode of action of discovered QTL alleles and extrapolation of QTL results to other, genetically linked individuals. In FruitBreedomics over 800 apple cultivars and breeding selections were SNP genotyped. The data allowed validation of historic pedigree records, tracing parentages of over 50 individuals for which pedigree records were lacking or false. This included major founding cultivars world-wide (e.g., ‘Delicious’) as well as ancestors from cultivars of specific interest (e.g., ‘Enterprise’; ‘Antonovka O.B’^47,53^. This new information considerably increased the usability of the existing, previously obscure genetic links among germplasm within and among breeding germplasm world-wide.

#### Explored genetic diversity in breeding programmes

In Europe, apple production has a narrow genetic base: half of the production comes from just four cultivars: ‘Golden Delicious’, ‘Gala‘, ‘Idared‘, ‘Red Delicious‘ (http://www.wapa-association.org/docs/2014/European_apple_and_pear_crop_forecast_2014_-_Summary.pdf). Moreover, the 20 most produced cultivars show close genetic relationships. This tendency towards mono-culture could be ‘vulnerable to a catastrophe’, e.g., in case a new disease would show up. In 1991, Way et al. ^54^ already alerted that ‘careful consideration of pedigrees and increased size of the genetic base are needed in future apple breeding strategies’. FruitBreedomics surveyed the genetic diversity that is currently used in apple breeding. Seventeen European programs participated, as well as programs from New Zealand and the USA. Over 3200 crosses and over 3000 ancestors were examined for their founder composition. Usually, programs build on a genetically narrow framework of a few founders. However, overall a major part of the genetic make-up of modern germplasm is of highly diverse origin coming from up to 90 founders within a single breeding program. The data document the huge efforts of breeders to include new traits from very distinct genetic resources, including wild species. The results will be useful to breeders and policymakers in the EU and worldwide to define an agenda on breeding strategies and research priorities related to genetic diversity.

### Genome-wide analysis studies (GWAS)

A work package of FruitBreedomics was dedicated to the study of germplasm. The objectives were to: (i) improve the knowledge of genetic variability in apple and peach collections by exploring the phenotypic and allelic diversity available in apple and peach germplasm collections (variability, population structure, LD) and defining potential national and European Core collections (CC); (ii) identify genomic regions contributing to the genetic control of horticultural traits through GWAS; (iii) supply breeders with tightly linked molecular markers for implementation into their MAB pipelines.

## Genome wide studies on the structure of the genetic diversity in apple and peach

Considering that a large number of apple and peach accessions had been maintained and evaluated for several traits and years in European repositories, we first surveyed and made available a list of the accessions existing within a number of key European genbanks (5477 apples and 2885 peaches), together with a short description of the traits and phenotypic records available, with their respective protocols. Common protocols and descriptors were also defined for both apple and peach for further phenotypic assessments. Afterwards subsets of accessions were selected for each collection (1560 apples and 1296 peaches) and their phenotypic records transferred to the FruitBreedomics database following the scales agreed. The peach collections considered were those maintained by the University of Milano, CRA (Italy), INRA-Avignon (France) and IRTA (Spain) plus a valuable Chinese collection representing a large portion of peach variability absent in the European germplasm collections (Zhejing University, China). The apple collections were those maintained by CRA-W-Gembloux (Belgium), INRA-Angers (France), RBIP-Holovouzy (Czech Republic), SLU-Balsgard (Sweden), University of Bologna (Italy), and NFC-Brogdale (UK). Phenotypic records for phenology, fruit quality, tree architecture, and some disease resistance data were uploaded to the FruitBreedomics database. Thereafter subsets of accessions were selected as representatives of the collections based on curators’ knowledge and/or available genotypic (SSR) data. This subset of accessions was phenotyped for key selected traits during at least two additional seasons in their respective location.

**Fig. 2:**
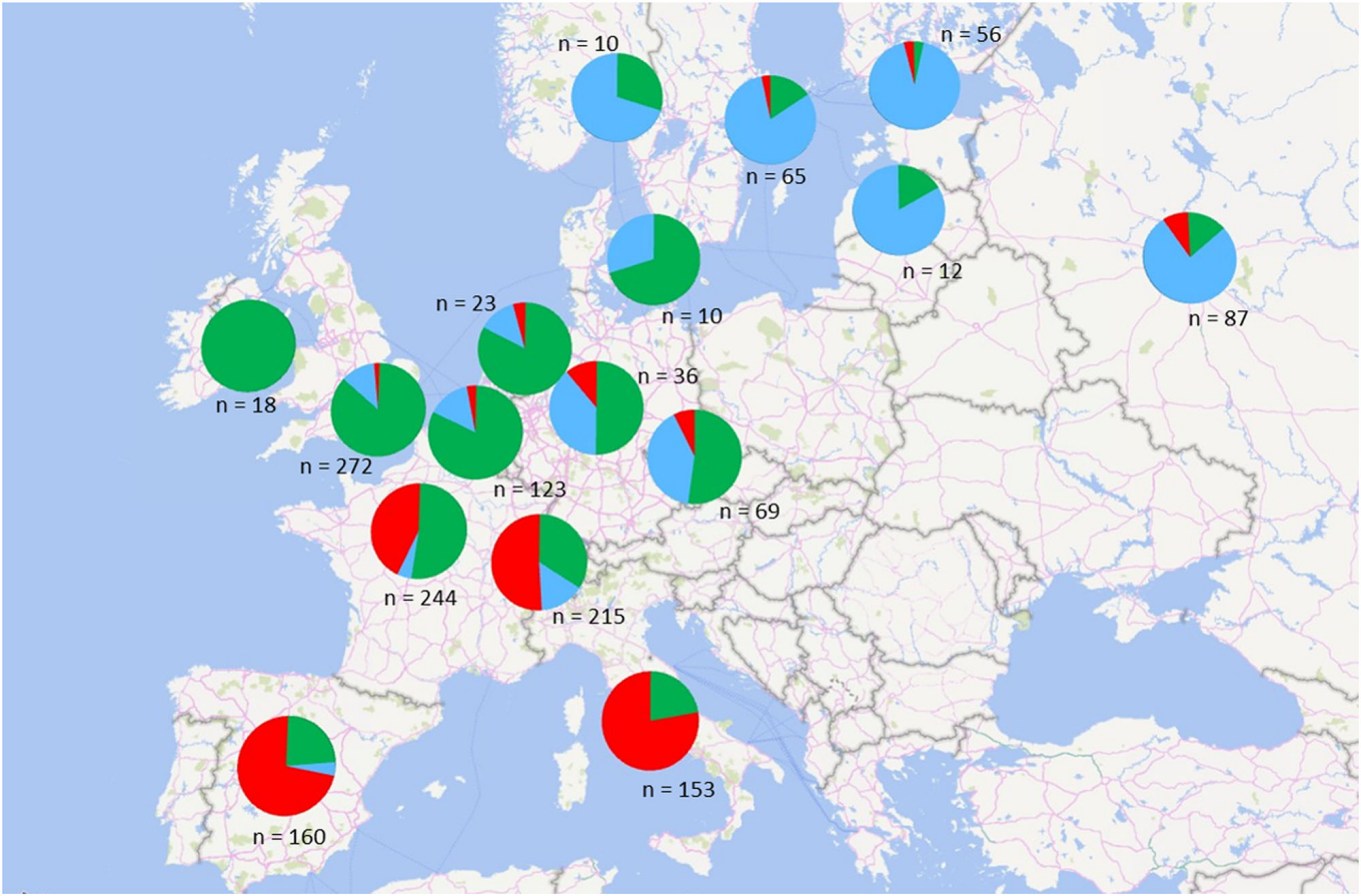
Genetic composition of the groups of apple cultivars clustered by country of origin for *K* = 3 groups (depicted in blue, green and red) inferred with Structure^58^. The pies represent the proportion of each country, with indication of the number of apple cultivars

Genotypic diversity was evaluated in both species. In several apple collections, a large fraction of the accessions had been previously genotyped with SSR markers^55–57^. Thanks to the FruitBreedomics project, a large number of additional apple accessions from various European germplasm collections were genotyped for diversity analysis with 16 common SSRs. Altogether, >2400 accessions were jointly analysed. The final sample included accessions from germplasm collections located in Belgium, France, Italy, United Kingdom, Czech Republic, Sweden, Switzerland, Spain, Russia and Kyrgyzstan. SSR data were used to identify synonyms and investigate the genetic structure of the whole gene pool, detecting differentiation according to the geographic origin of the accessions (West, South, North + East; Fig. 2). Overall, the apple genetic diversity was very high, but with a weak structure, confirming large gene flow across Europe^58^.

Genetic diversity of >1500 peach accessions was evaluated with the Illumina 9K peach SNP array. Data allowed the detection of synonyms, detecting 173 groups encompassing 473 cultivars with at least two identical cultivars (i.e., >98% of their SNPs genotypes identical); consequently 300 cultivars were removed. The 1240 unique peach accessions grouped in three main populations, which correlated with breeding strategies (breeding and local landraces) and also with geographic origin (Occidental and Oriental (Asia)^59^. SNP data also showed that most cultivars coming from modern breeding programs had an uneven distribution of homozygous and heterozygous regions along the genome, with large monomorphic regions followed by highly heterozygous regions. Given the high values of the kinship found (*k* = 0.58 on average), these results suggest that the monomorphic regions are identical by descent as a consequence of a high level of co-ancestry between peach cultivars.

Genotypic data together with phenotypic information obtained before and during FruitBreedomics allowed the definition of core collection (CC) representative for partner’s repositories as well as a first tentative European CC per species intended for future research studies. Such data have been extremely useful to identify clones and synonyms within and between collections, which will aid in a more efficient management of the apple and peach collections. These combined and co-ordinated datasets represent a significant development in the co-ordination of European genetic resource collections for both apple and peach, and will offer valuable base for continued efforts in bringing further germplasm collections together in the future.

**Fig. 3:**
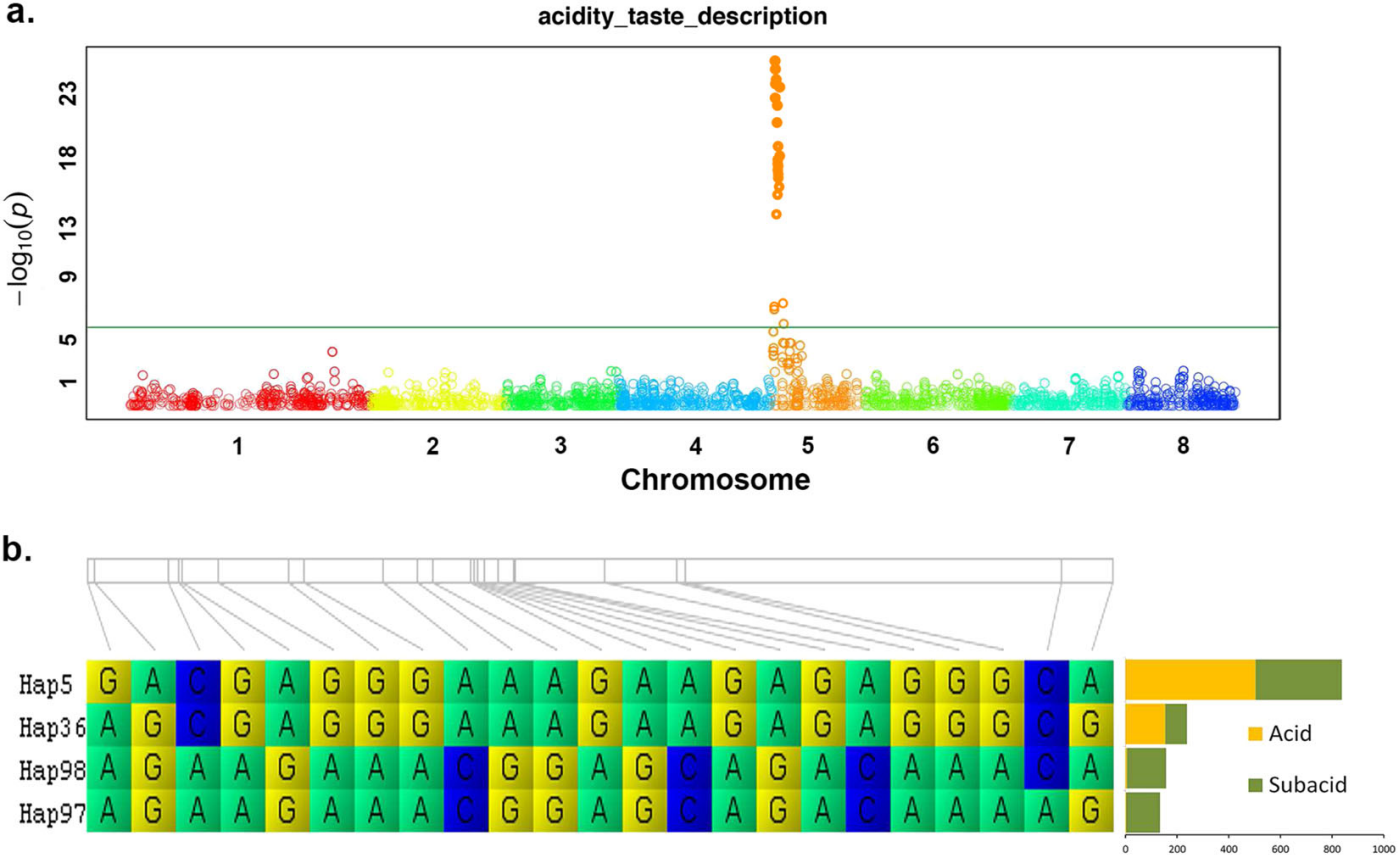
Genome wide association results for the acid/subacid trait in peach . **a** Manhattan plot. Chromosomes are marked with a different colour on the horizontal axis. The horizontal green line represents the significance threshold for the association. **b** Associated haplotypes constructed with the associated SNPs. The grey bar represents the position of the SNPs in a 1.8 Mb region. The bar chart represents the number of accessions with subacid and acid phenotype per haplotype

In peach, first GWAS analysis on monogenic traits validated the use of the SNP array for LD mapping^59^. A detailed analysis of the most frequent SNP haplotypes in European breeding programmes for the loci *G* (peach/nectarine), *Y* (white/yellow flesh), *S* (flat/round fruit shape) and *D* (acid/subacid fruits) allowed markers to be designed for MAB^60^. In general, the success in predicting the phenotype was high (>80%). As an example, Fig. 3a shows the Manhattan plot of the GWAS results for the acid/subacid trait in peach^59^. Twenty-three SNPs located at the top of chromosome 5 showed significant association with the trait. These SNPs were organized in 4 highly frequent haplotypes (Fig. 3b). Two were observed only in the subacid varieties (subacid is dominant over acid) and the two additional were observed in the acid varieties (when occurred in homozygosity) and in the subacid varieties (when occurred in heterozygosity).

For apple, GWAS analyses were also engaged by combining the previously described phenotypic data and SNP genotypic data newly gained thanks to the development of the Axiom_Apple480k array^22^. For phenological traits, highly significant genomic regions were identified on the top of chromosome 9 (flowering period) and on chromosomes 3, 10 and 16 (ripening period) ^23^, consistently with already published studies^14,61,62^.

## Improving the efficiency of fruit breeding programs

Based on the assessment made in 2010, the FruitBreedomics consortium decided to tackle the issue of the efficiency of breeding programs through two main ways, (1) improving and developing pre-breeding strategies, (2) implementing molecular markers in commercial breeding programs by making available the tools and genetic knowledge gained in the project.

### Prebreeding

To face the very narrow genetic basis FruitBreedomics aimed at the establishment of pre-breeding materials in apple and peach considering new traits in a more efficient way and further development and application in case studies of the early flowering approach in apple^63^.

Pre-breeding materials can be defined as materials carrying new traits or trait combinations, which can be used as donors to improve cultivar breeding by the introduction of these traits. The development of pre-breeding materials, a time-consuming and expensive process, is necessary if desired traits or combinations are not present in existing advanced breeding materials, i.e., if the donors are wild apple species or new combinations of desired traits contain too much genetic drag. Apple pre-breeding materials carrying pyramids of apple scab resistance or powdery mildew resistance genes or combinations of both have been developed by conventional breeding. Resistance genes *Rvi2*, *Rvi4*, *Rvi6*, *Pl1*, *Pl2*, *Pld* and *Plm* have been used for this process. Additionally, crosses were performed to introduce scab resistance from *M*. *baccata* jackii (*Rvi11*) and Hansens *baccata* No. 2 (*Rvi12*) as well as low chilling, low allergenic potential, specific aroma (litchi and mango flavour) and extraordinary fruit shape. The targets for peach were resistance to powdery mildew (*Vr1*, *Vr2*, SD-PM.6.1, SD-PM.8.1), green peach aphid *(Rm1*, *Rm2*, SD-MP.3.1, SD-MP.5.1) and brown rot; combined with specific traits involved in fruit quality such as slow ripening, non-acid flesh (D) and flat shape (S) as well as combinations of resistance to brown rot and powdery mildew. The efficiency of pre-breeding was increased by the choice of donors and the development of strategies to shorten the generation cycle in apple (fast-track approach). A list of crosses done and the availability of plant materials can be searched on the FruitBreedomics web page (www.fruitbreedomics.com).

The current early flowering approach in apple is based on the line T1190 which constitutively expresses the transcription factor *BpMADS4* inserted on the chromosome 4^64^. Genotypes carrying this ‘early flowering’ gene show slender shoots and their flowering time cannot be controlled. Therefore, within FruitBreedomics we tried to produced new lines using a heat shock promoter to induce flowering by the repression of TFL1 (terminate flower 1)^65^. Unfortunately, this approach failed, but the development of transgenic lines carrying the early flowering gene (*BpMADS4*) on different linkage groups was successful. *BpMADS4* lines carrying the flowering gene on linkage groups 2, 4, 5, 7, 8, 9, 10, 14, 15, 16 are now available and allow the application of the approach on traits also present on chromosome 4^66^.

The proof of concept of the early flowering approach clearly confirmed that using this approach one generation in around 1 year is possible. BC’4 plants carrying only the *FB_E* fire blight resistance locus from ‘Evereste’^67^ were developed in <7 years^68^ (note that 1 year was ‘lost’ due to technical problems) and for *FB_MR5*^69^ starting from an F1 hybrid in only four years. By classical breeding the production of the same generation would have taken 25 years or longer. Performing artificial inoculations with *E*. *amylovora* demonstrated that the final products, i.e., pre-breeding materials (at least BC’4) without the early flowering gene but carrying the fire blight resistance gene, possess a high level of fire blight resistance. Therefore, very valuable pre-breeding materials for the breeding of new fire blight resistant cultivars is available. In the USA such pre-breeding genotypes are not considered as genetically modified, US apple breeders can readily use them, but this chance will be precluded to European apple breeders if these genotypes will not be deregulated.

### Implementation of molecular markers in breeding programs

Another aim of FruitBreedomics was to develop more efficient breeding programmes applying marker assisted breeding. To reach this goal, the project aimed at designing and developing (i) molecular markers (SNPs) tightly linked to specific major horticultural traits, (ii) cost-efficient marker-assisted breeding (MAB) pipeline(s); and (iii) it also made a proof of concept of Genome Wide Selection on apple and peach.

#### Molecular assisted breeding

Prior to the practical development of MAB, the identification of SNP markers associated to specific apple and peach traits/loci started. The work led to the identification of several SNP markers. For apple, SNP markers were found for the apple scab resistance genes *Rvi2*, *Rvi4*, *Rvi5*, *Rvi6*, *Rvi11*, *Rvi12*, *Rvi13*, *Rvi15* and QTLs on LG17 of ‘Fiesta’ and ‘Discovery’, the fire blight resistance genes *FB_MR5*, *FB_E* and the QTL *FB_F7*, powdery mildew resistance gene *Pl2* and QTLs on LG 2 and 13 of TN10–8 and the rosy apple aphid resistance gene *Dp_Fl*^70,71^. For peach, SNP markers were found for fruit low acidity (locus *D*); fruit shape (flat/round, locus *S*) glabrous fruit epidermis (peach/nectarine locus *G*); fruit flesh colour (white/yellow locus *Y*), non-melting (locus *F*) and for the green peach aphid resistance gene *Rm2* and QTLs on LG3 and LG5 and finally for the powdery mildew resistance gene *Vr2* and QTLs on LG6 and LG8^60^.

Then a protocol reporting all the optimized steps necessary to perform MAB was developed together with a very efficient tracking system for seedlings avoiding the time consuming single labelling of each seedling. Moreover, a genotyping platform that can be used also by breeders without an own DNA lab was identified and tested in the apple and peach pilot studies. The apple and peach pilot studies had two aims: to validate the predictions done using the SNP markers, and to test the optimized MAB protocol and the selected genotyping platform. For the loci that could be validated, the predictions done with the SNP markers were correct in close or above 90% of the cases. The selection strategy tested in an apple pilot study allowed identifying 89 seedlings among over 5000 with pyramided resistance genes, best growth characteristics and potential best fruit quality traits (i.e. low ethylene production and best firmness^72^).

In the course of the project the steering committee realized that the development of SNP, the cost-effective markers, the optimization of a MAB protocol and identification of an efficient genotyping platform was not sufficient to facilitate the access of MAB of breeders that are willing to start applying molecular markers in their program. FruitBreedomics launched commercial ‘MAB Services’ to efficiently transfer MAB-related information to public and commercial Rosaceae breeding programs. The service was provided based on information gathered within FruitBreedomics and other relevant external sources.

#### Genome-wide selection

**Fig. 4:**
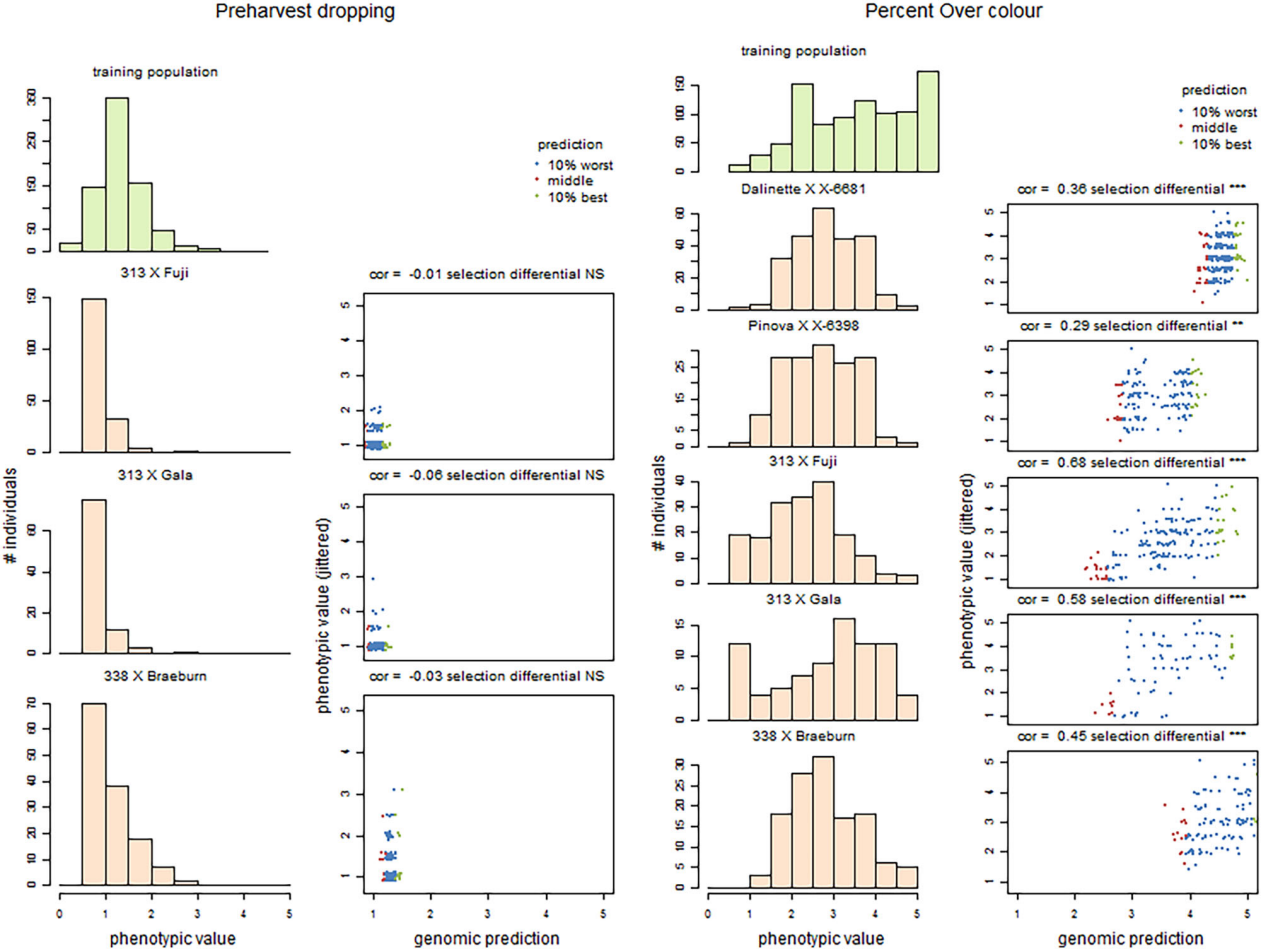
Within-training population distribution of genotypic BLUP (light green) used for building genome-wide prediction model in apple and within-application family distribution of phenotypic data (means over 2 years, beige) for two traits scored at harvest, Preharvest dropping (first column) and Percent Overcolour (third column) . Relationship between genomic predictions and phenotypic values for the same traits (Preharvest dropping: second column; Percent Overcolour: fourth column). The 10% (best) individuals with the highest predicted GBV are represented by green points, the 10% (worst) individuals with the lowest predicted GBV by red points, the other individuals by blue points. Accuracies, i.e. correlations between genomic predictions and phenotypic values, are written in the title line, followed by the significance of the difference between the 10% best and worst individuals

Genome wide selection (GWS) is a promising and innovative strategy to select among genotyped individuals when their phenotypes are not yet available, provided a related population was genotyped and phenotyped to estimate marker effects used for accurate prediction of breeding or genotypic value or phenotype. As for many other large perennials, this strategy is highly desirable for apple and peach breeding where phenotyping is lengthy, time-consuming and costly and concerns many traits. In FruitBreedomics project, an apple GWS experiment was pursued with the objectives to (1) assess the accuracy of prediction and (2) study realized response to selection. Twenty pedigree-related families, initially gathered for PBA, and their marker and phenotypic data, were used to build a genome-wide prediction model57. This model was applied to predict Genomic Breeding Values (GBV) in five full-sib families retrieved from commercial apple breeding programs of two FruitBreedomics breeders. These families were phenotyped over two consecutive years in breeders’ orchards and genotyped using TaqMan OpenArray plates preloaded with 512 SNPs uniformly distributed over the apple genome that were a subset of the 20K SNP of the Illumina array. SNP genotypes were completed through imputation up to the same number of SNPs as in the training population^73^. The accuracy of estimated genomic breeding values was estimated as the correlation between these predicted GBV’s and observed phenotypes. It varied largely among traits and among applications progenies and ranged between −0.31 and 0.68, with a median of 0.25. The accuracies were strongly affected by the phenotypic distribution and heritability of the traits. They were uncorrelated to the genomic relatedness of each family to the training population. Significant responses to genomic selection were obtained in 47 trait-family combinations out of 97, i.e., the top 10% and bottom 10% differed significantly. Figure 4 illustrates the dependency of accuracies to phenotypic distribution with two contrasting traits, pre-harvest dropping with highly asymmetric distributions in the three families where it was assessed, and percent over-colour with almost symmetric distributions^57^.

In peach, a set including >1100 seedlings (largely overlapping with those used for a parallel PBA study, see section ‘PBA’) was used as a basis to assess the feasibility of GWS for fruit weight, sugar content and titratable acidity. SNP data (9K IPSC array) were integrated with phenotypic data collected over 3–5 years in Italy, France, and Spain. Although results varied among different populations and traits, in general predictive abilities were greater than 60% on average^50^, indicating GWS as a promising approach in peach breeding programmes too.

## Conclusion and perspective

FruitBreedomics was a very important endeavour! It was an integrative project gathering 28 institutes and companies representing 14 countries and 367 stakehoders.

FruitBreedomics made significant breakthroughs in the development of tools, methodologies and knowledge for the fruit scientific community. The most significant ones are (i) the new genomic tools (20K and 487K apple SNP chips) which are the foundations of further genetic and genomic studies, (ii) softwares like ASSIsT, FlexQTL which helped identifying robust and informative molecular markers and building genetic maps, (iii) new discovery and fine mapping approaches like Pedigree-Based Analysis (PBA) and GWAS that allowed the discovery of additional molecular markers closely linked to important traits such as fruit quality and biotic and abiotic resistances to be used for genetic studies and marker assisted breeding.

The project also gave a deeper insight on the European apple and peach germplasm and breeding population structure that allows breeders to choose for new parent material to grasp new variability and introduce it into their breeding programs.

FruitBreedomics also provided the breeders with a huge number of pre-breeding materials and new phenotypic methods to assess traits of interest for apple and peach, in particular methods to assess fruit texture, resistance to *Monilinia* in peach and drought tolerance in apple. FruitBreedomics further developed pipelines for MAB on apple and peach.

Although FruitBreedomics ended in 2015 still with important results to be published, it has generated many short-term impacts for both fruit scientific and breeding communities. For example, peach resequencing data have generated a new add-on Illumina peach 18K SNP chip integrating ca 9000 SNPs in the already existing 9K IPSC chip. This new chip is currently being used by RosBreed partners for extensive genotyping. The decisive input of the dense SNP maps has widely applied for improving the quality of genome sequences for both apple and peach. Information on genetic diversity within European germplasm collections and breeding populations has helped to set up a new European experimental design on apple and peach to perform GWAS and genomic prediction with consideration of the Genotype × Environment × Management interaction. The first assessments will start in 2018.

Preliminary studies are ongoing to set up a common European Fruit Breeding Platform that will provide various practical services and commodities for breeders including an efficient breeding interface application to help breeders to better plan their crosses, new pre-breeding materials and MAB services.

## Electronic supplementary material

Sup Table 1(DOCX 17 kb)
