# Supplementary material for: An integrated approach for increasing breeding efficiency in apple and peach in Europe
Source: Hortic Res. 2018 Mar 1;5:11. doi: 10.1038/s41438-018-0016-3 (PMC5830435; doi:10.1038/s41438-018-0016-3)
Supplement: Supplementary file 1 — Sup Table 1(DOCX 17 kb) [file 41438_2018_16_MOESM1_ESM.docx]

**Sup Table1. List of the FruitBreedomics consortium participants**

| Participant name | short name | Country |
| --- | --- | --- |
| Institut National de la Recherche Agronomique | INRA | FRA |
| Agricultural Research Organisation | ARO | ISR |
| Agro Selection Fruits | ASF | FRA |
| Better3Fruit | B3F | BEL |
| Centre Wallon de recherches agronomiques | CRA-W | BEL |
| Consiglio per la Ricerca e la Sperimentazione in Agricoltura | CRA | ITA |
| Daniele Neri Vivai | DNV | ITA |
| Eidgenössische Technische Hochschule Zürich | ETHZ | CHE |
| Eidgenössisches Volkswirtschaftsdepartment | EVD | CHE |
| [Fundazione Edmund Mach](http://www.ismaa.it/) | FEM | ITA |
| Inra Transfert | IT | FRA |
| Institut de Recerca i Tecnologia Agroalimentàries | IRTA | ESP |
| Julius Kühn-Institut | JKI | DEU |
| Novadi S.A.R.L | Novadi | FRA |
| Parco Tecnologico Padano S.R.L | PTP | ITA |
| Research and Breeding Institute of Pomology Holovousy Ltd. | RBIPH | CZE |
| Rusticas del Guadalquivir | RDG | ESP |
| Stichting Dienst Landbouwkundig Onderzoek | DLO | NLD |
| Università degli Studi di Milano | UMIL | ITA |
| [Università di Bologna](http://www.unibo.it/) | UNIBO | ITA |
| University of Reading | Reading | GBR |
| Agricultural Research Council | ARC | ZAF |
| New Zealand Institute for Plant and Food Research Limited | PFR | NZL |
| Washington State University | WSU | USA |
| Zhejiang University  Katholische University of Leuven  Research Centre of Laimburg | ZJU  KUL  RCL | CHN  BEL  ITA |
